# High Prevalence of EMRSA-15 in Portuguese Public Buses: A Worrisome Finding

**DOI:** 10.1371/journal.pone.0017630

**Published:** 2011-03-02

**Authors:** Roméo Rocha Simões, Marta Aires-de-Sousa, Teresa Conceição, Filipa Antunes, Paulo Martins da Costa, Hermínia de Lencastre

**Affiliations:** 1 Laboratory of Molecular Genetics, Instituto de Tecnologia Química e Biológica, Oeiras, Portugal; 2 Instituto de Ciências Biomédicas de Abel Salazar, Universidade do Porto, Oporto, Portugal; 3 Escola Superior de Saúde da Cruz Vermelha Portuguesa, Lisbon, Portugal; 4 CIIMAR - Centro Interdisciplinar de Investigação Marinha e Ambiental do Porto, Oporto, Portugal; 5 Laboratory of Microbiology, The Rockefeller University, New York, New York, United States of America; National Institutes of Health, United States of America

## Abstract

**Background:**

The nosocomial prevalence of methicillin resistant *Staphylococcus aureus* (MRSA) in Portugal remains one of the highest in Europe and is currently around 50%. Transmission of *S. aureus*, including MRSA, occurs principally by direct human-to-human skin contact. However, *S. aureus* can survive for long periods on inanimate objects, which may represent an important reservoir for dissemination as well.

**Methodology/Principal Findings:**

Between May 2009 and February 2010, handrails of 85 public urban buses circulating in Oporto, Portugal, were screened for the occurrence of MRSA. Twenty-two (26%) buses showed MRSA contamination. The molecular characterization of a total of 55 MRSA, by pulsed-field gel electrophoresis (PFGE), staphylococcal cassette chromosome (SCC) *mec* typing, *spa* typing, and multilocus sequence typing (MLST), clustered the isolates into three clonal types. However, the overwhelming majority (n = 50; 91%) of the isolates belonged to a single clone (PFGE A, *spa* types t747, t032, t025 or t020, ST22, SCC*mec* type IVh) that exhibits the characteristics of the pandemic EMRSA-15, currently the major lineage circulating in Portuguese hospitals, namely in the Oporto region. Two additional clones were found but in much lower numbers: (i) PFGE B, ST5, *spa* type t002, SCC*mec* IVa (n = 3), and (ii) PFGE C, *spa* type t008, ST8, SCC*mec* IVa (n = 2). None of the 55 isolates was PVL positive.

**Conclusions/Significance:**

Public buses in Oporto seem to be an important reservoir of MRSA of nosocomial origin, providing evidence that the major hospital-associated MRSA clone in Portugal is escaping from the primary ecological niche of hospitals to the community environment. Infection control measures are urgently warranted to limit the spread of EMRSA-15 to the general population and future studies are required to assess the eventual increase of MRSA in the Portuguese community, which so far remains low.

## Introduction

Methicillin-resistant *Staphylococcus aureus* (MRSA) is one of the most important hospital-associated (HA-MRSA) pathogens, responsible for increased patient morbidity and mortality, length of hospitalization and higher healthcare costs. More recently, MRSA has emerged worldwide as a community-associated (CA-MRSA) pathogen, generating an additional public health concern. Although CA-MRSA are genetically different from nosocomial MRSA [1], the distinction between the two groups is blurring, since nowadays, CA-MRSA show multidrug resistance and are endemic in many hospitals [2], [3], [4], [5].

The nosocomial prevalence of MRSA in Europe varies considerably. The current prevalence of HA-MRSA in Portugal (49.1%) is among the highest in the continent (EARSS Annual report 2009 http://www.ecdc.europa.eu/en/publications/Publications/1011_SUR_annual_EARS_Net_2009.pdf). Considering this problematic situation in Portuguese hospitals, different investigations have been performed to evaluate the extension of CA-MRSA in the country. A first study in the late 1990s and another one a decade later, including isolates from nasal swabs of young healthy individuals and nasopharyngeal swabs of children attending day care centers, respectively, reported a prevalence of MRSA lower than 1% in the Portuguese healthy community [6], [7]. More recently, a study involving children attending the pediatric emergency department of a hospital due to skin and soft tissue infections, identified for the first time in Portugal a single CA-MRSA isolate ST80-IV producing the Panton Valentine leukocidin [8]. Therefore, although MRSA is a major problem in Portuguese hospitals, the prevalence of CA-MRSA, among children and youth, seems to remain low in the country.

Transmission of *S. aureus*, including MRSA, occurs principally by direct human-to-human skin contact. However, *S. aureus* can survive for long periods on inanimate objects, which may constitute an important reservoir for dissemination as well [9]. Therefore hand-touch surfaces in public transport vehicles, such as handrails, may represent a potential reservoir. Although methicillin susceptible *S. aureus* and methicillin resistant coagulase negative isolates have been found in public buses in London and Belgrade, respectively, MRSA was not detected in any of the studies [10], [11]. Nevertheless, ambulances [12], patient homes [13], public areas of hospitals [14], and environmental surfaces at emergency medical responders facilities [15] were found to represent possible reservoirs of MRSA.

The aim of the present study was to explore the extension of public buses as a reservoir of MRSA in Portugal, which shows the second highest prevalence of nosocomial MRSA in Europe. The molecular characterization of the isolates and comparison with the MRSA clones spread among Portuguese hospitals provided insights into the origin of the isolates.

## Materials and Methods

### Screened vehicles

Between May 2009 and February 2010, handrails of 85 public urban buses circulating in Oporto, Portugal, were screened for the occurrence of MRSA. The participating buses were assigned to 12 different lines/routes (Table 1). On average, the vehicles have 35 seating places and 90 standing ones. Superficial cleaning of the buses occurs every day but disinfection happens every three months only.

**Table 1:** Buses lines data and PFGE types found in the different vehicles contaminated with MRSA.

Bus line	Hospitals (b)	No. of buses contaminated with MRSA	PFGE types found in each contaminated bus(date of screening) (c)			Total number of MRSA isolates
1	1	3	A1 (16/06/2009)	A1 (16/06/2009)	A3 (27/10/2009)	8
2	1	1	A2 (19/01/2010)			1
3;7 (a)	2	1	A1 (23/09/2009)			1
4	0	1	A1 (11/11/2009)			3
5;9 (a)	1	1	A1 (24/11/2009)			3
6	1	1	A1 (11/02/2010)			3
7	1	2	A1 (16/06/2009)	A1, A6, C (11/11/2009)		8
8	2	3	A2 (11/11/2009)	A2 (11/11/2009)	A1, A4 (11/02/2010)	8
9	1	1	A2 (05/01/2010)			2
10	1	2	C (14/10/2009)	A1 (05/01/2010)		3
11	2	3	A1 (24/11/2009)	A5, B (05/01/2010)	A1 (11/02/2010)	10
12	2	1	A3 (05/01/2010)			3
12;7 (a)	3	2	A1 (16/06/2009)			2
12		22				55

(a) Two bus lines were assigned to the same vehicle during the screening day.

(b) Number of hospitals on each bus route.

(c) Each column corresponds to different buses assigned to the same bus line. Date of screening: day/month/year.

### Sampling and bacterial isolates

Samples were collected, soon after the vehicles had ended transportation and before any putative cleaning, using sterile gauzes moistened in Brain Heart Infusion broth - BHI (Oxoid, United Kingdom, Basingstoke) supplemented with 0.1% Tween 80 (Merck, Germany, Darmstadt). In each bus, a single gauze was used to sample a large surface of different handrails. Samples were kept for a maximum of two hours at 4°C in BHI broth until processing in the laboratory, where each sample was incubated at 37°C for approximately two hours. Subsequently, aliquots of 60 and 500 µ were inoculated onto three to five BBL™ CHROMagar™ Staph aureus plates (BD, NJ USA, Franklin Lakes), dried at 44°C for 10 minutes, and supplemented with 2 µg/ml of oxacillin. After 24 to 48 h of incubation at 37°C, all colonies exhibiting typical *S. aureus* morphology (mauve-colored colonies) were selected for antimicrobial susceptibility testing and storage. The plates with 500 µl of inoculum were incubated in an inverted position and partially open for the first 30 minutes. If any doubt subsisted concerning the species, isolates were tested for coagulase with a rapid latex agglutination test (BioMérieux, France, Marcy l'Etoile).

### Antimicrobial susceptibility testing

Antimicrobial susceptibility testing was performed by the disk diffusion method, following the Clinical and Laboratory Standards Institute guidelines [16] for a panel of 21 antimicrobial agents (Oxoid, United Kingdom, Basingstoke): oxacillin, ampicillin, cefoxitin, amoxicillin-clavulanic acid, ciprofloxacin, erythromycin, azithromycin, imipenem, kanamycin, tobramycin, gentamicin, quinupristin-dalfopristin, tetracycline, trimethoprim-sulfamethoxazole, rifampin, chloramphenicol, nitrofurantoin, clindamycin, linezolid, teicoplanin, and vancomycin. Methicillin resistance was confirmed on all isolates by PCR amplification of the *mecA* gene, as previously described [17].

### Pulsed-field gel electrophoresis

Pulsed-field gel electrophoresis (PFGE) was performed as previously described [18] on all MRSA isolates. The resulting *Sma*I restriction patterns were analysed by both visual inspection and computer analysis with Bionumerics version 6.1 software (Applied Maths, Sint-Martens-Latem, Belgium). Dendrogram was generated using an optimization of 0.5% and tolerance of 1.25% [19]. Similarity coefficients of 80% and 95% were used to define PFGE types and subtypes, respectively [19].

### *spa* typing, MLST and SCC*mec* typing

*spa* typing was performed on at least one representative isolate of each PFGE subtype, as previously described [20], and *spa* types were assigned through the Ridom web server (http://spaserver.ridom.de). Multilocus sequence typing (MLST) was carried out as described previously [21], on a representative isolate of each PFGE type. Allelic profiles and sequence types (ST) were assigned using the MLST database (http://www.mlst.net).

Staphylococcal cassette chromosome (SCC) *mec* was typed by the multiplex PCR strategy described by Milheiriço et al. [22] in each representative isolate of a different PFGE subtype-*spa* type association. Additionally, all SCC*mec* type IV isolates were subtyped as previously described [23].

### PVL detection

Panton Valentine leukocidin (PVL) detection was performed on all MRSA isolates according to a previous published protocol [24].

## Results and Discussion

**Figure 1:**
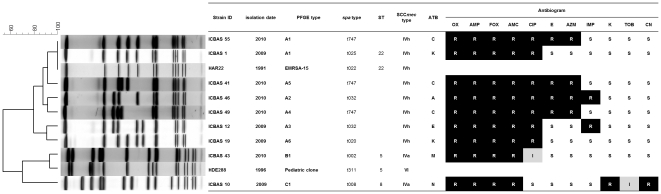
Characterization of representative MRSA isolates and comparison with MRSA pandemic clones. From left to right: (i) dendrogram, showing the estimated relationships of PFGE types based on Bionumerics analysis, including representatives of two international pandemic MRSA clones (EMRSA-15 and Pediatric clone); (ii) list of isolates; (iii) isolation date; (iv) PFGE type; (v) *spa* type; (vi) MLST sequence type (ST); (vii) SCC*mec* type; (viii) antibiotype (ATB); and (ix) antibiogram. R - resistance, I - intermediate susceptibility, S - susceptibility; Antibiotic abbreviations: OX – oxacillin, AMP – ampicillin, FOX – cefoxitin, AMC - amoxicillin-clavulanic acid, CIP – ciprofloxacin, E – erythromycin, AZM – azithromycin, IPM – imipenem, K – kanamycin, TOB – tobramicin, CN – gentamicin.

A total of 55 MRSA was recovered from 22 (26%) out of 85 buses. To our knowledge, this is the first study reporting MRSA in public urban buses. The molecular characterization of the 55 MRSA strains clustered the isolates into three clonal types (Figure 1). However, the overwhelming majority (n = 50; 91%) belonged to a single clone (PFGE A, *spa* types t747, t032, t025 or t020, ST22, SCC*mec* type IVh), which corresponds to the internationally disseminated EMRSA-15. In a study performed in 2006 in 11 Portuguese hospitals scattered all over the country, EMRSA-15 accounted for 54% of the total isolates [25]. The clone was detected in all hospitals and was the major lineage in seven of them, namely in the region of Oporto; EMRSA-15 represented 50% and 85% of the isolates in the two hospitals studied in Oporto and 82% in a hospital in Braga [25], a city located 50 km apart from Oporto. Moreover, a study performed in another hospital in Oporto showed that EMRSA-15 represented 79% of the isolates collected between 2003 and 2005 [26]. Recently, EMRSA-15 was also found as the prevalent clonal type (87%) in the Portuguese Azores islands [27]. The fact that 91% of the isolates recovered in the present study correspond to the major MRSA clonal type currently spread in all Portuguese hospitals [25], [27] is noteworthy and evidences the dissemination of a nosocomial MRSA clone from the hospital to the community environment. This situation may be explained, in part, by the fact that all but one bus lines included in the study pass near at least one hospital (Table 1), which in a country with a prevalence of nosocomial MRSA of 49.1% constitutes a plausible MRSA source. In addition, in Portugal, discharged patients are not decolonized for MRSA and screenings of health care workers colonization are not a routine practice. Hence, the contamination of the buses handrails most probably originated from the hands of health care workers, discharged patients, as well as outpatients and/or hospital visitors departing the hospitals.

Two additional clones were found in the present study but slightly represented, in agreement with the current situation in Portuguese hospitals: clone B - PFGE B, ST5, *spa* type t002, SCC*mec* IVa (n = 3; 6%) and clone C - PFGE C, *spa* type t008, ST8, SCC*mec* IVa (n = 2; 4%). Clone B is very similar to the Pediatric MRSA clone (Figure 1), which was described for the first time in a pediatric hospital in Lisbon [28]. Although the pediatric clone was a major clone in 1992–1997 in that particular hospital, it was detected in a single isolate only in the national study performed in 2006 [25]. Clonal type ST8-IV has been previously described mainly associated with CA-MRSA strains and in many cases coupled with PVL genes. In our study none of the 55 isolates was PVL positive.

PFGE subtype A1, the predominant (58%) subtype of PFGE type A, was found in all but one (n = 11) bus lines and during the whole study period (Table 1). The fact that the buses are only superficially cleaned every day, disinfected only every three months and that in a given day one to three different lines could be assigned to a same vehicle, may explain the fact that clonal type A1 is widely disseminated in this public transport network. Interestingly, in two cases, different PFGE types/subtypes were recovered in a same bus; PFGE types A1, A6 and C in one line 7 bus and PFGE types A5 and B in one line 11 bus (Table 1), evidencing the coexistence of different clonal types in a single vehicle.

Two previous studies have tried to demonstrate that public transports are a potential *S. aureus* reservoir, but both have failed to identify MRSA [10], [11]. The high salt concentrations in the selective media used in one of the studies were suggested to have inhibited the growth of MRSA [29]. In the present work, plating onto a chromogenic agar with a pre-enrichment step using a nonselective broth (BHI), might have contributed to the recovery of a significant number of MRSA isolates. Eventually, the screening could have been improved if using a semi-selective enrichment broth as described recently by Bocher and collaborators [30]. The second study published attributed the absence of MRSA in public buses to the low prevalence of MRSA carriage in the local healthy population [10]. However, in the present study public buses were found to constitute an important MRSA reservoir in Portugal despite the low MRSA colonization or infection in the community [6], [7]. Nevertheless, since the screenings for CA-MRSA were performed in the Southern region of Portugal (Lisbon and Montemor-o-Novo) future studies are required to assess the prevalence of EMRSA-15 among the general population in the region of Oporto where public buses were recently found to be highly contaminated with this MRSA lineage.

The smaller number of buses screened in the two previous studies, i.e. two buses in the London study [10] and 55 in the Belgrade work [11], may also be an explanation for the failure to identify MRSA. Additionally, a higher frequency of disinfection could also have contributed to the absence of MRSA. However, and although we do not possess information concerning the Belgrade buses, the London study reported that the surfaces sampled were infrequently cleaned sites [10]. EMRSA-15, the predominant clone highly spread in Oporto buses, harbors SCC*mec* type IV, one of the smaller cassettes, and consequently shows faster growth rates and resistance to a limited number of antimicrobial agents (Figure 1). Therefore, although it is a hospital-associated clone, EMRSA-15 shows similar traits to CA-MRSA that may favor its survival and persistence in the community. Although ST22-IV isolates had been previously found in the community in different animal species, particularly in dogs and cats, but also in turtles, bats and pet birds [31], and found to be able to circulate in human Australian remote communities [32], the first documented isolation of EMRSA-15 among healthy individuals with no HA-MRSA risk factors was only recently published from Ireland [33]. Consequently, the high prevalence of MRSA in Portuguese public buses might represent a first step for the massive spread of EMRSA-15 into the general population.

Allied to the fact that antimicrobial agents may still be bought over the counter in Portugal, the country shows one of the highest rates of outpatient antibiotic sales among the European Union [34], which may constitute sufficient selective pressure to maintain this MRSA clone in the community environment. In 2007, the annual outpatient antibiotics consumption in Portugal was estimated as 21.86 daily doses per 1000 people per day and the most used antibiotics were penicillins (52%), followed by macrolides (18%), quinolones (13%) and cefalosporins (10%) [35]. These correlated to the antimicrobial agents that showed decreased susceptibility among the isolates contaminating the buses in Oporto (Figure 1).

In addition, fat of hand sweat, warm temperatures, and appropriate humidity conditions observed in Portuguese buses circulating in the city of Oporto may also have played a role in the survival of *S. aureus* on the handrails of the vehicles. Tolba et al. demonstrated that *S. aureus* can easily be recovered from metallic surfaces and can survive for long periods in adequate conditions [36]. However, since the buses in Oporto circulate several times a day near at least one hospital, the MRSA isolates are probably being constantly (several times a day) inoculated on the handrails, which makes it difficult to distinguish between long time survival and recent inoculation.

In summary, public buses in Oporto seem to be an important reservoir of MRSA of nosocomial origin, providing evidence that the major hospital-associated MRSA clone in Portugal is escaping from the primary ecological niche of hospitals to the community environment. Consequently, infection control measures are urgently warranted to limit the spread of EMRSA-15 to the general population and future studies are required to assess the eventual increase of MRSA in the Portuguese community, which so far remains low.
